# Proceedings of the 2015 WAO Symposium on Food Allergy and the Microbiome

**DOI:** 10.1186/s40413-016-0097-0

**Published:** 2016-10-11

**Authors:** Raúl Lázaro Castro Almarales, Mary Carmen Reyes Zamora, Beatriz Tamargo, Damaris Torralba Averoff, Raysa Cruz, Yunia Oliva Diaz, Mirta Alvarez Castello, Alexander Ciria, Alexis Labrada, Maytee Mateo, Maytee Mateo, Damaris Torralba Averoff, Raysa Cruz, Yunia Oliva Diaz, Mirta Alvarez Castello, Alexander Ciria, Mary Carmen Reyes Zamora, Beatriz Tamargo, Alexis Labrada, Omar Herrera, Maytee Mateo, Raysa Cruz, Mirta Alvarez Castello, Alexander Ciria, Raúl Lázaro Castro Almarales, Mary Carmen Reyes Zamora, Alexis Labrada, José Severino Rodríguez Canosa, Raysa Cruz, Maytee Mateo, Mirta Alvarez Castello, Alexander Ciria, Raúl Lázaro Castro Almarales, Mary Carmen Reyes Zamora, Alexis Labrada, Mirta Alvarez Castello, Raúl Lázaro Castro Almarales, Alexis Labrada, Biocen, Yamilet Ibizate Novales, Ilonka Estruch Fajardo, Alexis Labrada, Maytee Mateo, Armando Ginard, Bruce Lanser, Anna Faino, Erwin Gelfand, Pia Hauk, Silvia Venero Fernández, Julia Urbina, Mirta Alvarez Castello, Raúl Lázaro Castro Almarales, Ramón Suárez Medina, Hermes Fundora Hernández, John Britton, Andrew William Fogarty, Nabarun Ghosh, Clinton Ross Bell, Chandini Revanna, Constantine Saadeh, Jeff Bennert, Danius Bouyi, Mitsy Veloz, Nelofar Sherali, Magna Coelho, Nabarun Ghosh, Jeff Bennert, Danius Bouyi, Constantine Saadeh, Clinton Ross Bell, Mitsy Veloz, Chandini Revanna, Nelofar Sherali, Joseph J. Dolence, Takao Kobayashi, Koji Iijima, Hirohito Kita, Hirohito Kita, Ashli Moore, James Krempski, Roberta Aina, Riccardo Asero, Sabine Pfeifer, Pawel Dubiela, Merima Bublin, Christian Radauer, Piotr Humeniuk, Karin Hoffmann-Sommergruber, Frank Eidelman, Ves Dimov, Charl Khalil, Ves Dimov, Frank Eidelman, Charl Khalil, Alice E. W. Hoyt, Peter Heymann, Alexander Schuyler, Scott Commins, Thomas Platts-Mills, Alexander Schuyler, Patrice Kruszewski, John Russo, Lisa Workman, Thomas Platts-Mills, Elizabeth Erwin, Anubha Tripathi, Gabriela Yvette Castellanos, Elizabeth Mendieta, Martín Becerril-Angeles

**Affiliations:** 1 National Center of Bioproducts, Bejucal, Cuba; 2 Institute of Pharmacy and Foods, Havana, Cuba; 3 University Hospital Calixto García, ᅟ, Cuba; 4 Hospital William Soler, Aldabo, Cuba; 5 National Center of Bioproducts, ᅟ, Cuba; 6 University Hospital Calixto García, Havana, Cuba; 7 Hospital William Soler, ᅟ, Cuba; 8 Institute of Pharmacy and Foods, ᅟ, Cuba; 9 University Hospital Calixto Garcia, ᅟ, Cuba; 10 National Center of Bioproducts, ᅟ, Cuba; 11 Hospital William Soler, ᅟ, Cuba; 12 Cuban Society of Immunology, Cuban Society of Allergy, Asthma and Clinical Immunology, ᅟ, Cuba; 13 National Center of Bioproducts, ᅟ, Cuba; 14 University Hospital Calixto García, ᅟ, Cuba; 15 Hospital William Soler, ᅟ, Cuba; 16 University Hospital Calixto García, ᅟ, Cuba; 17 National Center of Bioproducts, ᅟ, Cuba; 18 Biocen, ᅟ, Cuba; 19 Pediatric Hospital “Pepe Portilla”, ᅟ, Cuba; 20 University Hospital Hermanos Ameijeiras, ᅟ, Cuba; 21 National Center of Bioproducts, ᅟ, Cuba; 22 Biocen, ᅟ, Cuba; 23 National Jewish Health, ᅟ, CO USA; 24 National Institute of Hygiene Epidemiology and Microbiology, ᅟ, Cuba; 25 Health Direction Municipality La Lisa, ᅟ, Cuba; 26 University Hospital Calixto García, ᅟ, Cuba; 27 National Center of Bioproducts, ᅟ, Cuba; 28 University of Nottingham, Nottingham, UK; 29 West Texas a&m University, ᅟ, TX USA; 30 Allergy Arts, Amarillo, TX USA; 31 Texas Tech University, Lubbock, TX USA; 32 Air Oasis, ᅟ, TX USA; 33 Unimontes, ᅟ, Brazil; 34 West Texas a&m University, ᅟ, TX USA; 35 Air Oasis, ᅟ, TX USA; 36 Allergy Arts, Amarillo, TX USA; 37 Texas Tech University, Lubbock, TX USA; 38 Mayo Clinic College of Medicine, ᅟ, MN USA; 39 Mayo Graduate School, ᅟ, MN USA; 40 Medical University of Vienna, ᅟ, Austria; 41 Clinica San Carlo, ᅟ, Italy; 42 Cleveland Clinic Florida, ᅟ, USA; 43 Cleveland Clinic, Florida, USA; 44 University of Virginia, ᅟ, USA; 45 University of Virginia, ᅟ, USA; 46 Emory University, ᅟ, USA; 47 Nationwide Children’s Hospital, ᅟ, USA; 48 Mexican Institute of Social Security, ᅟ, Mexico

## Abstract

A1 Characterization of the immunoallergic profile towards the proteins of the wheat flour in Cuban population

Raúl Lázaro Castro Almarales, Mary Carmen Reyes Zamora, Beatriz Tamargo, Damaris Torralba Averoff, Raysa Cruz, Yunia Oliva Diaz, Mirta Alvarez Castello, Alexander Ciria, Alexis Labrada, Maytee Mateo

A2 Are peanuts causing food allergy in Cuba?

Maytee Mateo, Damaris Torralba Averoff, Raysa Cruz, Yunia Oliva Diaz, Mirta Alvarez Castello, Alexander Ciria, Mary Carmen Reyes Zamora, Beatriz Tamargo, Alexis Labrada

A3 Prick test and immunoallergic profile to soy allergens in Cuban population

Omar Herrera, Maytee Mateo, Raysa Cruz, Mirta Alvarez Castello, Alexander Ciria, Raúl Lázaro Castro Almarales, Mary Carmen Reyes Zamora, Alexis Labrada

A4 Skin sensitization and immunoallergic profile to hen's egg in Cuban population

José Severino Rodríguez Canosa, Raysa Cruz, Maytee Mateo, Mirta Alvarez Castello, Alexander Ciria, Raúl Lázaro Castro Almarales, Mary Carmen Reyes Zamora, Alexis Labrada

A5 Sensitization to three domestic mites in patients with adverse food events to shellfish

Mirta Alvarez Castello, Raúl Lázaro Castro Almarales, Alexis Labrada, Biocen

A6 Diagnostic efficacy by skin prick test with allergenic extracts of legumes in Cuban patients

Yamilet Ibizate Novales, Ilonka Estruch Fajardo, Alexis Labrada, Maytee Mateo, Armando Ginard

A7 Baked egg goods without wheat flour carry an increased risk of reaction

Bruce Lanser, Anna Faino, Erwin Gelfand, Pia Hauk

A8 Prevalence, incidence and associated risk factors of adverse reaction to food in Cuban infants - a population-based prospective study

Silvia Venero Fernández, Julia Urbina, Mirta Alvarez Castello, Raúl Lázaro Castro Almarales, Ramón Suárez Medina, Hermes Fundora Hernández, John Britton, Andrew William Fogarty

A9 Microbiome in ice machines and assessing the plasma nanotechnology in breaking the biofilm and improving air quality

Nabarun Ghosh, Clinton Ross Bell, Chandini Revanna, Constantine Saadeh, Jeff Bennert, Danius Bouyi, Mitsy Veloz, Nelofar Sherali

A10 Characteristics of patients with food allergy in health public service

Magna Coelho

A11 Allergic rhinitis and asthma index increased in Texas panhandle and AHPCO and plasma nanotechnology as solutions

Nabarun Ghosh, Jeff Bennert, Danius Bouyi, Constantine Saadeh, Clinton Ross Bell, Mitsy Veloz, Chandini Revanna, Nelofar Sherali

A12 Antigen-specific T follicular helper cells mediate peanut allergy in mice

Joseph J. Dolence, Takao Kobayashi, Koji Iijima, Hirohito Kita, Hirohito Kita, Ashli Moore, James Krempski

A13 Production of recombinant Mal d 3, a major apple allergen, in Pichia Pastoris, to investigate the impact of the food matrix and post-translational modifications on Mal d 3 immuno-reactivity

Roberta Aina, Riccardo Asero, Sabine Pfeifer, Pawel Dubiela, Merima Bublin, Christian Radauer, Piotr Humeniuk, Karin Hoffmann-Sommergruber

A14 Reaction to sports drink: no whey! Whey allergy in absence of clinical cow’s milk allergy

Frank Eidelman, Ves Dimov, Charl Khalil

A15 Food allergy on Tumblr: focus on teenage audience may increase educational impact

Ves Dimov, Frank Eidelman, Charl Khalil

A16 Changes in IgE levels following one-year immunizations in two children with food allergy

Alice E. W. Hoyt, Peter Heymann, Alexander Schuyler, Scott Commins, Thomas Platts-Mills

A17 IgE and IgG4 antibodies to cow's milk components in children with eosinophilic esophagitis: higher specific IgG4 antibodies and IgG4:IgE ratios compared with subjects with IgE-mediated food allergy

Alexander Schuyler, Patrice Kruszewski, John Russo, Lisa Workman, Thomas Platts-Mills, Elizabeth Erwin, Anubha Tripathi

A18 Frequency of Sensitization to Food Allergens in Patients with Rhinitis and Asthma in the National Medical Center La Raza “Dr. Antonio Fraga Mouret”, Mexico City

Gabriela Yvette Castellanos, Elizabeth Mendieta, Martín Becerril-Angeles

## A1 Characterization of the immunoallergic profile towards the proteins of the wheat flour in Cuban population

### Raúl Lázaro Castro Almarales^1^, Mary Carmen Reyes Zamora^1^, Beatriz Tamargo^2^, Damaris Torralba Averoff^1^, Raysa Cruz^1^, Yunia Oliva Diaz^1^, Mirta Alvarez Castello^3^, Alexander Ciria^4^, Alexis Labrada^1^, Maytee Mateo^1^

#### ^1^National Center of Bioproducts, Bejucal, Cuba; ^2^Institute of Pharmacy and Foods, Havana, Cuba; ^3^University Hospital Calixto García, Cuba; ^4^Hospital William Soler, Cuba

**Introduction:** Wheat is recognized as an important source of food allergens. Wheat flour is one of the most consumed food in Cuba, even from very early ages. However, there is little data about the degree of immunoallergic reaction to wheat flour in the Cuban population. The aim of this study was to characterize the immunoallergic response towards the proteins of the wheat flour in Cuban patients.

**Materials and methods:** Four groups were analyzed: non-allergic adults, patients with symptoms of allergy to wheat flour (adults and children) and a highly exposed population (bakers) population samples. Sensitization to allergen extract was determined by skin prick tests and serum specific IgE and IgG4 antibodies which were identified different protein components of the extract, using the technique of Western blotting.

**Results:** The positivity rate of skin test for allergic adults was 54.4% and lower, 47.1% for allergic children. Prevalence among bakers was higher (34.1%) than in the general population. The most frequent clinical diseases were asthma and rhinitis. Eight proteins were identified by IgE and IgG4 antibodies. Among the allergenic proteins recognized Tri 30, and a protein of 65 kDa that could correspond with Tri 19. The recognition profile of IgG4 antibodies was broader than IgE but generally included this.

**Conclusion:** This study suggests that allergy to wheat flour in the Cuban population, could be more important than expected and similar to published dates in some industrialized countries.

## A2 Are peanuts causing food allergy in Cuba?

### Maytee Mateo^1^, Damaris Torralba Averoff^1^, Raysa Cruz^1^, Yunia Oliva Diaz^1^, Mirta Alvarez Castello^2^, Alexander Ciria^3^, Mary Carmen Reyes Zamora^1^, Beatriz Tamargo^4^, Alexis Labrada^1^

#### ^1^National Center of Bioproducts, Cuba; ^2^University Hospital Calixto García, Cuba; ^3^Hospital William Soler, Cuba; ^4^Institute of Pharmacy and Foods, Cuba

**Introduction:** Prevalence of food allergies increase worldwide. Peanut (*Arachis hypogaea*) is a major cause of IgE-mediated reactions. However, in Cuba, sensitization to peanut allergens has not been properly studied. Therefore, the objective of this study was to assess the relevance of peanuts as food allergens in Cuba and to characterize the immunoallergic response in this population.

**Materials and methods:** Non-allergic and allergic patients (including adults and children) were selected. Skin Prick tests were performed using allergen extracts of raw and roasted peanut. Sensitization was also assessed by testing IgE and IgG4 serum antibodies specific to different extract components by Western blotting.

**Results:** The prevalence of sensitization in non-allergic adult population was 2.6% for raw and 4.6% for roasted peanuts. The maximum values were recorded in allergic children: 26% for raw and 23% for roasted peanut. The most common clinical manifestations were urticaria or allergic dermatitis in children and asthma or rhinitis in adults. Correlation between size of skin reactions to raw and roasted peanut was high (r = 0.698). Four major proteins with IgE and IgG4 binding activity were identified, among them, putatively, the allergens Ara h1 (60kDa) and Ara h3 (64kDa). The binding profile of IgG4 antibodies was broader than IgE but generally included it.

**Conclusion:** This study provides data showing relatively high values of allergic sensitization to peanuts in Cuba, close to those reported in first-world countries. Allergenic proteins recognized by Cubans largely coincide with allergens identified internationally. This study points to the need to develop more research on food allergies in our country.

## A3 Prick test and immunoallergic profile to soy allergens in Cuban population

### Omar Herrera^1^, Maytee Mateo^2^, Raysa Cruz^2^, Mirta Alvarez Castello^1^, Alexander Ciria^3^, Raúl Lázaro Castro Almarales^2^, Mary Carmen Reyes Zamora^2^, Alexis Labrada^2^

#### ^1^University Hospital Calixto Garcia, Cuba; ^2^National Center of Bioproducts, Cuba; ^3^Hospital William Soler, Cuba

**Introduction:** Food allergy is increasing worldwide. Soy is widely used as a hidden ingredient in many food products. Sensitization to Soy allergens can be important in Cuba, although the exact sensitization prevalence is not well known.

**Objective:** To perform a preliminary assessment of allergic sensitization and IgE specificity profile to Soy allergens in Cuban allergic patients.

**Methods:** The Skin Prick Test was performed using a glycerinated allergenic extract of soy beans (BIOCEN, Cuba) at 5 mg/mL protein content. Two groups were studied: a cohort of general adult population (N = 303), and patients suspected of food allergy, attending allergy services at 4 hospitals in Havana, comprising 159 adults (above 16 years old) and 157 children (2-16 years). A group of 40 bakers exposed to soy flour was also studied. The IgE and IgG4 binding profile of 32 selected SPT-positive patients, was further analyzed by Western Blotting.

**Results:** In the general adult cohort the prevalence of sensitization was 4.3%. Whereas, among patients suspecting food allergy the positivity rate was much higher: 68.2% for adults and 34.4% for children. The most frequent clinical manifestation among positive patients was Urticaria or Dermatitis (69%) for adults and for children respiratory symptoms were also common (74%). 24.4% of bakers were SPT-positive, showing mostly respiratory symptoms. IgE binding was mostly shown by a 30KDa band in bakers (possibly Gly m Bd30K, a cysteine-protease). The IgG_4_binding profile was similar to IgE.

**Conclusions:** Allergic sensitization to soy as food can be important in Cuba, with prevalence values similar to other countries. Therefore, it should be taken into account for improving the specific diagnosis of allergy.

## A4 Skin sensitization and immunoallergic profile to hen's egg in Cuban population

### José Severino Rodríguez Canosa^1^, Raysa Cruz^2^, Maytee Mateo^2^, Mirta Alvarez Castello^3^, Alexander Ciria^4^, Raúl Lázaro Castro Almarales^2^, Mary Carmen Reyes Zamora^2^, Alexis Labrada^2^

#### ^1^Cuban Society of Immunology, Cuban Society of Allergy, Asthma and Clinical Immunology, Cuba; ^2^National Center of Bioproducts, Cuba; ^3^University Hospital Calixto García, Cuba; ^4^Hospital William Soler, Cuba

**Introduction:** Food allergy is increasing worldwide. Allergy to hen’s egg can be important, particularly among children, and is relatively common in Cuba, although the exact sensitization prevalence is not well known.

**Objective:** To perform a preliminary assessment of allergic sensitization and IgE specificity profile to hen’s egg white allergens in Cuban allergic patients.

**Methods:** The Skin Prick Test was performed to each patient/subject, using a glycerinated allergenic extract, prepared in BIOCEN, Cuba, at 5 mg/mL protein content. Two groups of subjects were studied: a cohort of general adult population (N = 303), and patients attending allergy services at 4 hospitals in Havana, comprising 159 adults (above 16 years old) and 157 children (5-16 years). The IgE and IgG_4_ binding profile of 20 selected SPT positive patients, was further analyzed by Western Blotting.

**Results:** In the general adult cohort the prevalence of sensitization was 3.6%. Whereas, among patients suspecting food allergy the positivity rate was much higher: 39.6% for adults and 36.3% for children. The most frequent clinical manifestation among positive patients was Urticaria or Dermatitis (62-67%), although respiratory symptoms were also common (40-49%). IgE binding was shown mostly by the 45KDa band, tentatively identified as Gal d 2, a known major allergen of clinical importance. Also, the 77KDa band (probably Gal d 3) showed binding in 2 patients. The IgG4 binding profile was similar to IgE, although with some additional bands.

**Conclusions:** Allergic sensitization to egg can be important in Cuba, with prevalence values similar to other countries. Therefore, it should be taken into account for improving the specific diagnosis of allergy.

## A5 Sensitization to three domestic mites in patients with adverse food events to shellfish

### Mirta Alvarez Castello^1^, Raúl Lázaro Castro Almarales^2^, Alexis Labrada^3^, Biocen

#### ^1^University Hospital Calixto García, Cuba; ^2^National Center of Bioproducts, Cuba; ^3^Biocen, Cuba

**Background:** Food allergy to shellfish affects a large population all over the world. It is well established that most allergic patients to shellfish present sensitization to mites, as well. The aim of this study was to assess the sensitization to three species of house dust mites in adults with food allergy to shellfish.

**Method:** A descriptive and cross-sectional study was carried out. The study included 25 adult patients with allergic reactions to food shellfish. Mean age: 37 (range 22-62 years). Six cases had occupational exposure, and one of them had food-dependent exercise-induced anaphylaxis. For each subject, a clinical and occupational history was compiled and skin testing was performed. Skin Prick Test (SPT) was performed with standardized allergen extracts (BIOCEN, Cuba) of Dermatophagoides pteronyssinus, D. siboney and Blomia tropicalis at 20 000 BU/mL.

**Results:** 100% subjects showed a positive response to Dermatophagoides mites by SPT. Twenty subjects had personal history of atopy (80%). Shrimp was the most common seafood involved. The most reported symptoms after food consumption were cutaneous (84%) followed by gastrointestinal (76 %) and respiratory symptoms (60 %), mostly, dyspnea. The largest mean wheal size was reported to Blomia tropicalis (5.82mm), followed by D. siboney (4.97mm). 100 % of patients that were occupationally exposed showed positive response to shrimp and lobster. A positive and strong correlation was found between reaction size to shrimp and lobster.

**Conclusion:** There is a high prevalence of sensitization to mites in adults with allergic reactions to shellfish. The co-existent atopic disease, sensitization to mites and occupational exposure are factors to consider in food allergy to shellfish.

## A6 Diagnostic efficacy by skin prick test with allergenic extracts of legumes in Cuban patients

### Yamilet Ibizate Novales^1^, Ilonka Estruch Fajardo^2^, Alexis Labrada^3^, Maytee Mateo^4^, Armando Ginard^2^

#### ^1^Pediatric Hospital “Pepe Portilla”, Cuba; ^2^University Hospital Hermanos Ameijeiras, Cuba; ^3^National Center of Bioproducts, Cuba; ^4^Biocen, Cuba

**Background:** In many countries legumes are a very common basic food. Immediate hypersensitivity to legumes is increasing worldwide. Allergenic extracts with proper diagnostic efficacy and safety are required for performing skin prick tests (SPT). The purpose of this study was to evaluate the diagnostic efficacy and safety of locally manufactured legume extracts (peanut, soy and black bean) by means of the SPT in Cuban patients.

**Methods:** A study was carried in thirty seven patients with immediate clinical history to peanut, soy and/or black bean that attended the Allergy Service at the Hospital Hermanos Ameijeiras in Havana between 2010 and 2011. SPT was performed with extracts of raw soy, raw and cooked black bean (at 2,5mg/ml protein content) and raw and roasted peanut (0,5mg/ml) prepared by the National Center of Bioproducts (BIOCEN, Cuba). It was carried a double-blind, placebo-controlled food challenge (DBPCFC) to 20 patients, using progressively growing doses.

**Results:** Using a wheal diameter of 3 mm as cutoff value, the SPT showed a diagnostic efficacy of 73.0% (soy), 70.3% (raw black bean), 81.1% (cooked black bean), 67.6% (raw peanut) and 75.7% (roasted peanut) taking as a reference of the disease the clinical history. As compared to DBPCFC, the SPT for soy and peanut had sensitivity of 100% and for the bean, of 67%. There were not serious reactions.

**Conclusions:** The allergenic extracts used for SPT are effective and safe for the diagnosis of food allergy. The methodology for the DBPCFCs is safe, but it is recommended to increase the maximum dose.

## A7 Baked egg goods without wheat flour carry an increased risk of reaction

### Bruce Lanser, Anna Faino, Erwin Gelfand, Pia Hauk

#### National Jewish Health, CO, USA

**Rationale:** Eating egg protein in baked form has been shown to hasten outgrowing of an egg allergy. Baking egg with wheat flour decreases in vitro antigenic activity to heat-resistant ovomucoid. This is believed to be due to the “matrix effect,” wherein the sequential epitope is polymerized and forms high-molecular weight complexes. To study the matrix effect and the importance of wheat flour on baked egg tolerance in vivo, we examined the outcomes of OFCs to baked egg in egg allergic children.

**Methods:** A 2-year retrospective chart review was performed in 104 egg allergic children, ages 0.9 to 16.8 y (mean 5.7 y), who were sensitized to egg by skin test and/or specific IgE and who underwent OFCs to baked egg. The effect of wheat flour or a wheat replacer (rice flour) on OFCs to baked egg in a standardized muffin (2.2 g of egg protein) was assessed.

**Results:** Eighty-nine (85.6%) children were challenged with a muffin baked with wheat flour. Fifteen (14.4%) received a muffin containing wheat replacer. Overall, 68 (65.4%) children passed and 36 (34.6%) failed OFCs to baked egg. In the wheat group, only 30.3% (27/89) failed, while 60% (9/15) of the non-wheat group failed OFCs to baked egg. Females comprised only 37% of the cohort. After adjusting for age, gender, and history of atopic dermatitis, the odds ratio of failing an OFC to baked egg with a muffin containing wheat replacer was 5.3 (95% CI 1.54,18.2; p = 0.0083) compared to a muffin containing wheat flour.

**Conclusions:** Children undergoing OFCs to egg in baked goods made with wheat replacer may be at an increased risk for failing an OFC. Wheat replacers should only be used when clinically indicated, as in wheat allergic children. Children who pass a baked egg OFC using a muffin made with wheat flour should be warned that they might be at risk for reacting to baked egg products made without wheat.

## A8 Prevalence, incidence and associated risk factors of adverse reaction to food in Cuban infants - a population-based prospective study

### Silvia Venero Fernández^1^, Julia Urbina^2^, Mirta Alvarez Castello^3^, Raúl Lázaro Castro Almarales^4^, Ramón Suárez Medina^1^, Hermes Fundora Hernández^1^, John Britton^5^, Andrew William Fogarty^5^

#### ^1^National Institute of Hygiene Epidemiology and Microbiology, Cuba; ^2^Health Direction Municipality La Lisa, Cuba; ^3^University Hospital Calixto García, Cuba; ^4^National Center of Bioproducts, Cuba; ^5^University of Nottingham, Nottingham, UK

**Background**: The paediatric population is known to have high rates of asthma, eczema and allergic rhinitis, but little is known about food allergy and intolerance. The objective was determining prevalence, incidence and risk factors of Adverse reaction to food in paediatric population.

**Methods**: A random sample of 1543 children aged 3 years old living in Havana was included in the data base of study Natural History of Wheezing in a Cohort of Cuban Infants (HINASIC). Data were collected using questionnaires administered by researchers. Adverse reaction to food was defined by medical diagnosis reported by parents. Nutritional status of mother during pregnancy was defined by Cuban charts for this group. The data were analysed by dichotomous logistic regression using a step-wise modelling approach. The study protocol was approved by National Institute of Hygiene, Epidemiology and Microbiology Ethical and the Scientific Committee Havana in Cuba and also by the University of Nottingham Medical School Ethics Committee in the United Kingdom. The oral and written consent was obtained from the baseline by the legal guardians of children.

**Results:** The prevalence of adverse reaction to any food were 5.7%, 4,0% and 2,5% at age 1, 2 and 3 years respectively. The annual incidence of new food sensitivity in the previous year was 5.7%, 1.9% and 0.8% at age 1, 2 and 3 years respectively. The most common food involved was cow milk (44.6 %). Frequent systems affected are gastrointestinal tract and skin. The most important associated risk factors were: consumption of allergenic foods (OR: 2.33; 95% confidence intervals CI: 1.28-4.25), use of antibiotics (OR: 1.88; CI: 1.09-3.23), factors related to presence of environmental allergens such as cocking in child room (OR: 1.75; CI: 1.24-2.47) and mold at home (OR:1.65; CI: 1.18-2.31), maternal history of overweight defined by Cuban standard for nutritional evaluation of weight during pregnancy (OR: 1.64; CI: 1.08-2.49) and insect sting allergy (OR: 1.55; CI: 1.05-2.29).

**Conclusions**: Adverse reaction to food is a significant clinical problem in a Cuban paediatric population. Modifiable risk factors were identified, although it is unclear if these associations are causal. Better understanding of these associations will help inform the implementation of effective intervention strategies in the future.

## A9 Microbiome in ice machines and assessing the plasma nanotechnology in breaking the biofilm and improving air quality

### Nabarun Ghosh^1^, Clinton Ross Bell^2^, Chandini Revanna^3^, Constantine Saadeh^2^, Jeff Bennert^4^, Danius Bouyi^1^, Mitsy Veloz^1^, Nelofar Sherali^1^

#### ^1^West Texas a&m University, TX, USA; ^2^Allergy Arts, Amarillo, TX, USA; ^3^Texas Tech University, Lubbock, TX, USA; ^4^Air Oasis, TX, USA

Bacteria that adhere to any equipment can encase themselves in a hydrated matrix of polysaccharide and protein, and form a slimy layer known as a biofilm. Once microbes grow into well-developed biofilms, cleaning and sanitation become difficult. With the advent of moisture and organic media biofilms are formed on the walls of the ice makers and refrigerators. Scientific tests revealed that ice from many restaurants had higher levels of bacteria than samples of water taken from their lavatory bowls. Dirty ice machine causing contamination via ice cubes is also a major health problem in other countries including the United States^1^. To prevent any potential contamination, the interior surface biofilm microflora in the ice machine must be sanitized regularly. We have analyzed the microbiome from an ice machine in a local restaurant in Amarillo, Texas. The swab-culture showed Gram positive and negative *Bacilli*, *Penicillium chrysogenum, Alternaria alternata* conidia*, Pithomyces* sp. spores in the swab samples collected from the upper wall and the floor of the ice maker. We evaluated the Plasma Nanotechnology applied in the Bi-Polar unit in sanitizing the ice machine surface. The unit creates cold plasma discharge which consists of positive and negative ions from water vapor in the air. Positive and negative ions attach to particles which cluster together to create inactivated larger particles. Two sets of petri-plates were inoculated with sterile cotton swab with the inoculum collected from the ice-maker surface at the time intervals of 24, 48, 72, 120 and 168 hours. Developed colonies were observed after 24 hours of incubation at 37^o^ Celsius. Bacterial/fungal colonies were isolated using a SZ-40 stereo-scope. Prepared slides from bacterial colonies stained with Gram staining and fungi with Lacto-Phenol Cotton Blue stain were observed and micrographed at 100X with a Leica DM-750 microscope. The plotted graph showed a gradual reduction in microflora in the collected swab-cultures on using the sterilizing Bi-Polar unit while the composition and concentration of the microflora remain constant in the control set. After running the unit 168 hours, there was a significant reduction in microbial entities. This technology will prove to be an efficient way of reducing the contaminants in ice and indoor aeroallergens^2^.

**References**

1. Dirty Ice-Maker in Houston, Texas: http://www.houstontx.gov/health/Food/slime.htm

2. Ghosh, N., M. Veloz, J. Bennert D. Bouyi, and C. (2015) New World of Business with Plasma and AHPCO Nano-Technology in Marketing Air Purifier, Cell Phone and Ice-Maker Sterilizer. Book of Abstracts. International Journal of the Computer, the Internet, and Management (ISSN: 0858-7027) Sec. OST-13.P. 33. IMPACT, Thailand, 3-4 June, 2015

## A10 Characteristics of patients with food allergy in health public service

### Magna Coelho

#### Unimontes, Brazil

**Background:** Food allergy is an important public health problem that affects children and adults and may be increasing in prevalence. Here we want to characterize the most frequent cases of food allergy that was made diagnostic in the new clinic of reference in allergy of the ambulatorial specialty center in a medium-sized city, important in attendance in public health.

**Methods:** Cross-sectional study, approved by the ethics committee from CAAE 39525514.0.0000.5146, which included 200 patients treated at allergy service in the period from February to December 2013. Based on the consultations, we selected 16 patients involved in the diagnosis only of food allergy to be characterized. The instrument was a questionnaire prepared with this purpose and the data were collected through registers of medical records in which were evaluated some variables, including age, national origin, gender, chief complaint, clinical and laboratory diagnosis, and personal and family history of food allergies.

**Results:** The majority of patients with food allergy were male, corresponding to 10 patients (62.5%). The age group with the highest prevalence occurred in early childhood from 0 to 9 years (81.3%). The positivity was confirmed by skin prick test and serum specific IgE. Much of the observed complaints were rash, under the most common forms of clinical presentation for flushing, pruritus, urticaria, angioedema. The foods most commonly found to cause food allergies were cow's milk (37.5%) and egg white (12.5%). The patients were medicated and guided to specific nutritional caution and frequent return.

**Conclusions:** The prevalence of food allergy in our service was higher in the pediatric population and the main foods liable for these allergies were cow's milk and egg white.

## A11 Allergic rhinitis and asthma index increased in Texas panhandle and AHPCO and plasma nanotechnology as solutions

### Nabarun Ghosh^1^, Jeff Bennert^2^, Danius Bouyi^1^, Constantine Saadeh^3^, Clinton Ross Bell^3^, Mitsy Veloz^1^, Chandini Revanna^4^, Nelofar Sherali^1^

#### ^1^West Texas a&m University, TX, USA; ^2^Air Oasis, TX, USA; ^3^Allergy Arts, Amarillo, TX, USA; ^4^Texas Tech University, Lubbock, TX, USA

Global warming is accelerating slowly and that has exerted significant impact on the biotic system. Warmer years resulted into a gradual shift in flowering seasons and many plant species triggered more pollen production to ensure the survival in the changed global climate. The aeroallergen data that we collected using a Burkard Spore Trap for 15 years showed a steady increase in aeroallergen concentration in the Texas Panhandle area. Frequently trapped aeroallergens were: Pollen: *Ambrosia artemisiifolia*, *Helianthus ciliaris*, *H. hirsutus*, *H. annuus*, *Chenopodium album*, *Pinus sylvestris*, *Solanum elaeagnifolium, S. rostratum*; Fungal spores: *Curvularia*, *Cladosporium*, *Drechslera*, Ascospores, Teliospore and *Alternaria alternata*conidia. A strong correlation was found between the allergen index and allergy and asthma cases that have doubled since 2007. Even the slightest increase in average annual temperature caused a dramatic increase in average annual pollen count in Texas Panhandle in the past 15 years. Clinical data collected from the AARTS clinic showed that there were more patients suffering from allergic rhinitis during the months of May to August in 2007 and 2008. The peak aeroallergen season was gradually shifted and extended to April to September in 2009-11 and so as the frequency of the patients. A gradual shift in the aeroallergen index and that caused the increased cases of allergic rhinitis. The slow but steady increase in average temperature, dramatic shift in flowering season, excessive pollen production due to warmer climate led to doubling the rate of asthma cases in Amarillo in Texas Panhandle since 2007. The gradual shift in aeroallergen index with the warmer climate and a shift in flowering seasons were noticed that contributed the increased allergy cases. A collaborative research has developed a novel AHPCO or Advanced Hydrated Photocatalytic Oxidation technology to produce filter less air purifiers, surface sterilizer for cell phones and this technology can be used in meat processing facilities and in the ice makers to reduce the chances of contamination. The AHPCO and Plasma nanotechnology were successfully implemented in making the Air Purifiers, Surface Sterilizer and Ice-Maker Sterilizers. We assessed the efficiency of these air purification units that showed significant reduction in indoor aeroallergens including fungal and bacterial spores including MRSA, VOC, animal dander and particulate matters. Plasma nanotechnology has been used to prevent contamination in ice-makers and during food processing. The Plasma Nanotechnology was used in Bi-Polar units that exhibited significant reduction in microbial entities including bacteria, fungi, slime molds and Cyanobacteria.

## A12 Antigen-specific T follicular helper cells mediate peanut allergy in mice

### Joseph J. Dolence^1^, Takao Kobayashi^1^, Koji Iijima^1^, Hirohito Kita^1,2^, Hirohito Kita^1,2^, Ashli Moore^1,2^, James Krempski^1,2^

#### ^1^Mayo Clinic College of Medicine, MN, USA; ^2^Mayo Graduate School, MN, USA

**Background:** Peanut (PN) allergy is a widespread and life-threatening medical problem. The prevalence of the disease has been increasing. However, our knowledge of the mechanisms involved in the development of peanut allergy is extremely limited. Several major questions remain including the route of peanut allergen sensitization and the cells and molecules involved in the sensitization process. The goal of this project was to use mouse models and fill these major gaps in our knowledge.

**Methods:** We administered peanut (PN) flour intranasally (i.n.) to BALB/c (once/week) and C57Bl/6 (twice/week) for 4-weeks; no adjuvants were used. Mice were then challenged by intraperitoneal (i.p.) injection of PN extract. Development of anaphylactic response was monitored by rectal temperature and clinical symptom scores. Development of PN-specific Th2 cells and T follicular helper (Tfh) cells was analyzed by using the IL-4-IRES-eGFP (4get) reporter mouse. Role of Tfh cells were examined by using CD4-cre + Bcl6^fl/fl^mice, which are deficient in Tfh-specific transcription factor Bcl6.

**Results:** Intranasal exposure of mice to peanut flour sensitized naïve BALB/c and C57Bl/6 mice without any additional adjuvants, and these mice developed PN-specific IgE and IgG1 antibodies. When mice were challenged i.p. with PN extract, they developed anaphylactic responses, including lower body temperature and several clinical signs (e.g. rubbing, labored breathing, slowed motility) consistent with anaphylaxis. When exposed to PN flour, 4get mice developed Tfh cells (CD4+ CXCR5+ ST2- IL-4eGPP+), but few Th2 cells (CD4+ CXCR5- ST2+ IL-4eGFP+). Tfh-deficient mice failed to produce PN-specific IgE and IgG antibodies and they were protected from developing anaphylaxis upon PN challenge.

**Conclusion**: These findings suggest that allergen sensitization to PN can occur by airborne exposure. Furthermore, Tfh cells, but unlikely Th2 cells, play a major role in development of IgE antibodies and clinical outcomes. Significance of Tfh cells as compared to conventional Th2 cells in various allergic diseases need to be evaluated in future.

## A13 Production of recombinant Mal d 3, a major apple allergen, in Pichia Pastoris, to investigate the impact of the food matrix and post-translational modifications on Mal d 3 immuno-reactivity

### Roberta Aina^1^, Riccardo Asero^2^, Sabine Pfeifer^1^, Pawel Dubiela^1^, Merima Bublin^1^, Christian Radauer^1^, Piotr Humeniuk^1^, Karin Hoffmann-Sommergruber^1^

#### ^1^Medical University of Vienna, Austria; ^2^Clinica San Carlo, Italy

**Background:** Non-specific lipid transfer proteins (nsLTPs) are important plant food allergens able to induce severe systemic reactions in sensitized individuals. The aim of this project is to evaluate the impact of food matrix interaction and post-translational modifications on the allergenicity of nsLTPs, studying apple as a model food and its nsLTP, Mal d 3, as model food allergen. Notably, to perform these studies, a high amount of well characterized and biologically active allergen is necessary. Therefore, our first goal is to produce rMal d 3 in the yeast *Pichia pastoris,*which has been shown to be an efficient system for expressing huge amount of soluble and immunologically active nsLTPs.

**Methods:** The DNA sequence of mature Mal d 3 was codon optimized for *P. pastoris,* cloned into the pPICZαA vector and propagated in *E. coli* cells. The linearized pPICZαA-Mal d 3 plasmid was used to transform *P. pastoris* cells. Positive transformants were selected and the presence of the insert analyzed by PCR. Selected clones were cultured and screened for the expression of the recombinant protein by SDS-PAGE and immunoblotting with rabbit antiserum against nsLTP and allergic patients’ sera. rMal d 3 was purified from the culture supernatant by chromatographic methods (IEC and RP-HPLC) and analyzed by MALDI-TOF mass spectrometry.

**Results:** Multy-copy *P. pastoris* clones (n = 12) were selected, cultured and analyzed. Immunoblotting results showed that recombinant proteins retain both IgG- and IgE-binding capabilities. Two clones*,* highly expressing rMal d 3, were selected to perform a large scale production of the recombinant allergen. Purified rMal d 3 migrates in SDS-PAGE as a double band between 10 and 15 kDa. MALDI-TOF MS analysis confirmed the identity of the purified recombinant proteins, providing 9,553 kDa and 9,752 kDa (corresponding to the calculated theoretical masses of 9,560 kDa and 9,761 kDa, respectively), which resulted from different cleavage site of the signal sequence. Both forms were recognized by both anti-nsLTP antiserum and allergic patients’ sera.

**Conclusions:** Our data confirm the suitability of *P. pastoris* for the expression of nsLTPs. Purified rMal d 3 will be used to perform specific experiments to evaluate the impact of selected food matrix components (pectins, lipids..) and PTMs on its allergenicity. Supported by Marie-Curie project CARAMEL 626572.

## A14 Reaction to sports drink: no whey! Whey allergy in absence of clinical cow's milk allergy

### Frank Eidelman, Ves Dimov, Charl Khalil

#### Cleveland Clinic Florida, USA

**Introduction:** Allergy to cow’s milk is common and is associated with allergen-specific IgE directed against both caseins and whey proteins. Worldwide sales of sports related protein products grew from $3.9 billion in 2007 to $7.6 billion in 2012 and are likely to reach $12.2 billion in 2017. Whey and soy are the dominant protein ingredients in sports food. A case of allergy to a whey protein supplement in the absence of clinical allergy to cow’s milk is reported here.

**Case presentation:** A 9-year-old male who had a prior history of food allergy (milk, egg, peanut, sesame) became skin test negative after age 5 and was able to consume these foods. He presented to the ER with facial swelling, generalized urticaria, pruritus, vomiting and tremor after he drank a whey protein drink following a soccer practice. Allergy skin test for foods (including milk) performed 4 weeks later were negative. Whey specific IgE level was positive at 0.93 kU/L. The patient avoided whey protein drinks but continued to drink milk with no symptoms. More than 1 year later, he presented to the emergency department with diffuse hives and angioedema after consuming a large amount of milk before and after a soccer practice. That reaction was likely related to the amount of whey, as he was able to consume regular amounts of milk since then.

**Discussion:** Whey proteins are normal constituents of milk that are separated from caseins during the production of cheese. Whey is a mixture of proteins which are commonly used in a variety of products (global whey protein market $5.4 billion 2014). Previously reported whey protein allergens include beta-lactoglobulin, alpha-lactalbumin, immunoglobulins, and albumin. Clinical allergy to whey protein has been previously reported in the context of cow’s milk allergy. It is more common in young children and tends to wane with age. The case illustrates that hypersensitivity to whey proteins may elicit allergic reactions when whey is consumed in concentrated form despite the lack of clinical cow’s milk allergy.

**Conclusion:** Whey protein powder allergy can occur in the absence of cow’s milk allergy. Considering the increase in protein sport drink consumption, clinicians should be aware of that occurrence. Analysis of the clinical presentation and the relevance of the available sIgE whey protein components ( Alpha-lactalbumin (f76) IgE, Beta-lactoglobulin (f77) IgE, Casein (f78) IgE) could be a focus of future research studies.

## A15 Food allergy on Tumblr: focus on teenage audience may increase educational impact

### Ves Dimov, Frank Eidelman, Charl Khalil

#### Cleveland Clinic Florida, USA

**Purpose:** Tumblr is a micro-blogging platform and social networking website that was launched in 2007. As of August 1, 2015, Tumblr hosts over 420 million users, 248 million blogs, 117 billion total posts, and 74 million daily posts on average. This study aimed to determine the presence of food allergy-focused journals, organizations, groups and popular individual accounts on Tumblr.

**Methods:** The top 10 most popular allergy/immunology journals were identified from SCImago online service, www.scimagojr.com. The popularity of professional and and patient centered food allergy organizations, groups and individuals was ranked according to Tumblr algorithm and Google search engine. “Food allergy Tumblr” was searched on Google to determine the first five distinct results that were active within the last six months.

**Results:** None of the top 10 allergy/immunology journals maintained a Tumblr blog. Only Food Allergy Research and Education (FARE) organization maintained an active blog on Tumblr that targeted teenager readers, called “Food Allergy Teens”. Four of the first five Tumblr blogs on Google were maintained by layman individuals, not by professionals.

**Conclusion:** Despite its widespread popularity, Tumblr remains a social media domain that lacks a strong presence from allergy/immunology journals, food allergy organizations and allergists/immunologists. This represents an untapped resource for information dissemination and interaction with the public. It could provide an avenue to reach different demographic populations with the potential for dynamic education and possible life/disease-changing impact.

## A16 Changes in IgE levels following one-year immunizations in two children with food allergy

### Alice E. W. Hoyt, Peter Heymann, Alexander Schuyler, Scott Commins, Thomas Platts-Mills

#### University of Virginia, USA

**Background:** As food allergy has increased, so has the number of immunizations containing alum given to children. Knowing that alum contributes to the stimulation of IgE in mice, we question the impact of alum adjuvant on IgE production in children.

**Methods:** Two unrelated children presented to the Pediatric Allergy Clinic: an 8 month-old girl (Case 1) and a 12 month-old boy (Case 2).

Case 1: At 5 months of age, the otherwise healthy girl tasted peanut butter and developed hives and facial swelling within 20 minutes. Total and food allergen-specific IgE values were measured by ImmunoCAP 250 at ages (months) 8, 12, 12.7.

Case 2: At 8 months old, the otherwise healthy boy developed diffuse hives after eating scrambled egg. His total and food allergen-specific IgE values were measured at ages (months) 12 and 12.7.

**Results:** Case 1: At 8 months-old, her serum IgE values (in IU/mL) were total 61.4, peanut 13.6, almond 4.04, milk 3.84, egg 2.01, soy 1.6, and wheat 0.98, compared to total 44.1, peanut 11.2, almond 1.54, milk 2, egg 1.71, soy 1.62, and wheat 2.2 at 12 months-old. After the 12 month-old labs were drawn, she received the vaccines Prevnar13, hepatitis A, MMR, and Varicella. 3 weeks later, at 12.7 months-old, her IgE values were total 75.6, peanut 16.5, almond 2.18, milk 5.06, egg 3.4, soy 3.64, and wheat 3.75.

Case 2: At 12 months-old, the boy’s IgE values were total 21.1, egg 1.16, and peanut, milk, wheat, soy, cod, and shrimp <0.1. A week later, he received Prevnar13, MMR, and Varicella. 3 weeks later, at 13 months-old, his IgE values were total 23.8, egg 4.02, and peanut, milk, wheat, soy, cod, and shrimp <0.35 (measured by ImmunoCAP 1000).

**Conclusions:** We report two cases of children who were sensitized to food allergens whose serum IgE levels increased after immunizations. Case 1 shows total IgE and food allergen-specific IgE values that decreased from 8-12 months of age, a time interval during which she received no intramuscular alum. Three weeks after she received four vaccines (two of which contained alum), all of her IgE levels increased. In Case 2, the boy received three vaccines (one of which contained alum), and his total and egg IgE increased. The results raise the hypothesis that alum-containing vaccines may increase, at least temporarily, the production of allergen-specific IgE levels.

## A17 IgE and IgG4 antibodies to cow's milk components in children with eosinophilic esophagitis: higher specific IgG4 antibodies and IgG4:IgE ratios compared with subjects with IgE-mediated food allergy

### Alexander Schuyler^1^, Patrice Kruszewski^2^, John Russo^3^, Lisa Workman^1^, Thomas Platts-Mills^1^, Elizabeth Erwin^3^, Anubha Tripathi^1^

#### ^1^University of Virginia, USA; ^2^Emory University, USA; ^3^Nationwide Children’s Hospital, USA

**Background:** Eosinophilic esophagitis (EoE) in adults is characterized by elevated serum IgG4 antibodies (Ab) to whole food allergens with low to negative specific IgE Ab. The mechanistic involvement of IgG4 Ab in EoE is unclear because these Ab not only are predominately bispecific *in vivo* and not known to elicit inflammation via Fc binding, but also their appearance is a marker of tolerance during oral immunotherapy (OIT). Presented is an analysis of specific IgG and IgE Ab to cow’s milk components in children with EoE and to other relevant allergen components in subjects with IgE-mediated food allergy.

**Methods:** Sera were assayed for specific IgE and IgG4 Ab to cow’s milk components Bos d 4, Bos d 5, Bos d 6 and Bos d 8, galactose-alpha-1,3-galactose (alpha-gal), and/or peanut component Ara h 2 by ImmunoCAP. Specific pan-IgG and IgG4 Ab to Bos d 5 or alpha-gal were measured by adsorbing serum Ab onto solid-phase Protein G-Sepharose and anti-IgG4-Sepharose respectively, followed by incubation with radiolabeled allergen. Levels of serum IgG1 (and IgG2) Ab to Bos d 5 and alpha-gal and specific IgG4:IgE ratios were determined.

**Results:** IgG4 Ab to Bos d 4, Bos d 5, and Bos d 8 were at least 5-fold higher, and specific IgG4:IgE ratios were greater than 100-fold higher in children with EoE as compared with subjects with delayed anaphylaxis to red meat, or peanut anaphylaxis before or after OIT (p < 0.001). Although there was no obvious association between histological remission (<15 eosinophils/hpf) and serology following treatment (i.e., swallowed steroids or cow’s milk elimination diet) in children with EoE, a reduction in serum IgG4 Ab to cow’s milk components and specific IgG4:IgE ratios was observed among those treated with cow’s milk elimination diet. Focused serology revealed IgG1 and IgG4 Ab to Bos d 5 with low to negative levels of IgE Ab to cow’s milk components in the sera of children with EoE. In contrast, the antibody response in subjects with delayed anaphylaxis to red meat was dominated by IgE and IgG1 Ab to alpha-gal with low to undetectable levels of IgG4 Ab.

**Conclusions:** Specific IgG4:IgE ratios to cow’s milk components are very high in children with EoE, and this may explain the lack of positive results for cow’s milk components using the Immuno Solid-phase Allergen Chip (ISAC) microarray test. Our results are in keeping with a model where the pathology of EoE is not due to allergen-specific IgE Ab, and is more likely to be T cell-mediated.

## A18 Frequency of Sensitization to Food Allergens in Patients with Rhinitis and Asthma in the National Medical Center La Raza “Dr. Antonio Fraga Mouret”, Mexico City

### Gabriela Yvette Castellanos, Elizabeth Mendieta, Martín Becerril-Angeles

#### Mexican Institute of Social Security, Mexico

**Background:** The allergic respiratory diseases such as rhinitis and asthma are common in children and have a negative impact on the quality of life this patients, causing high costs in terms of health care. The estimated prevalence of food allergy in European studies is 25% of school children with allergic diseases; so it is necessary to determine the frequency of sensitization to food allergens in pediatric patients with allergic rhinitis and asthma in Mexico.

**Methods:** We conducted a transversal, descriptive, observational study to patients affiliated to Allergy department, in a third level Hospital "Dr. Antonio Fraga Mouret, "Mexico City. We included 100 patients (69 men and 31 women) from 3 to 18 years with rhinitis and asthma. We divide them in three age groups: 3 to 5 ; 6 to 10 ; and 11 to 18 years. We made to each one medical record , skin prick tests to aeroallergens and food.

**Results:** The average age of patients was 9.05 years, 69% were men. The most common aeroallergens were dust mite, ash tree, cat, oak tree, rye grass and tumbleweed. In this study we found positive skin test to food antigens in 45%. The average food was 3.1 per patient. The group that showed a higher percentage of food sensitization was 6 to 10 years (24%) predominantly soybeans, egg, shrimp, chicken, tuna and pumpkin, the second group was the 11-18 years (16%) positive for beans, gilthead sea bream, shrimp, red snapper, sea bass; and children under 5 years finding soybeans, egg and peanuts in 5%.

**Conclusions:** Food sensitization in patients with allergic rhinitis and asthma is common in the Mexican population, the most frequently identified were soy foods, egg, shrimp; which predominated in all age groups. Peanuts , egg , soy beans shows importance in children under 5 years and the fish meat, shirmp in adolescents.

